# Dual Action of *Pueraria montana* var. *lobata* Extract on Myogenesis and Muscle Atrophy

**DOI:** 10.3390/nu17071217

**Published:** 2025-03-30

**Authors:** So Young Eun, Chang Hoon Lee, Yoon-Hee Cheon, Chong Hyuk Chung, Myeung Su Lee, Ju-Young Kim

**Affiliations:** 1 Musculoskeletal and Immune Disease Research Institute, School of Medicine, Wonkwang University, 460 Iksandae-ro, Iksan 54538, Republic of Korea; eunsoyg@hanmail.net (S.Y.E.); lch110@wku.ac.kr (C.H.L.); hanleuni@naver.com (Y.-H.C.); taylorchung@hanmail.net (C.H.C.); 2 Department of Pharmacology, School of Medicine, Wonkwang University, 460 Iksandae-ro, Iksan 54538, Republic of Korea; 3 Division of Rheumatology, Department of Internal Medicine, Wonkwang University Hospital, 460 Iksandae-ro, Iksan 54538, Republic of Korea

**Keywords:** *Pueraria montana* var. *lobata* extract, muscle atrophy, protein synthesis, mitochondrial biogenesis, dexamethasone

## Abstract

**Background/Objectives**: Muscle atrophy, defined by diminished muscle mass and function, is a notable concern associated with aging, disease, and glucocorticoid treatment. *Pueraria montana* var. *lobata* extract (PMLE) demonstrates multiple bioactive properties, such as antioxidant, anti-inflammatory, and metabolic regulatory activities; however, its role in muscle atrophy has not been extensively investigated to date. This study examined how PMLE influences both muscle cell differentiation and dexamethasone (DEX)-induced muscle degeneration by focusing on the underlying molecular mechanisms. **Methods**: This study examined the effects of PMLE on myogenic differentiation and DEX-induced muscle atrophy. C2C12 myoblasts were treated with PMLE (10–100 ng/mL) and assessed for changes in the expression of myogenesis-related genes and activation of Akt/mTOR and AMPK/SIRT1/PGC-1α signaling cascades. In vivo, a DEX-induced muscle atrophy model was used to assess muscle mass, fiber morphology, and molecular changes. **Results**: PMLE PMLE promoted muscle cell development by increasing the expression of MyHC, MyoD, and myogenin while activating protein synthesis and mitochondrial biogenesis pathways. PMLE counteracted DEX-induced myotube atrophy, restoring myotube diameter and promoting cellular fusion in vitro. In vivo, PMLE mitigated muscle degradation in fast-twitch muscle groups and reversed DEX-induced suppression of key anabolic and mitochondrial pathways. **Conclusions**: These findings suggest that PMLE promotes myogenic differentiation and protects against muscle atrophy by regulating critical molecular pathways, indicating its promise as a treatment candidate for conditions involving muscle wasting. Further studies are required to assess its clinical application and long-term safety efficacy.

## 1. Introduction

Skeletal muscle atrophy is characterized by the gradual reduction in muscle mass and strength, significantly increasing the risk of physical disability and reduced quality of life, particularly in aging populations [1]. While consistent physical activity and proper nutrition can help preserve muscle integrity, factors such as aging, genetic background, and chronic illnesses can still lead to muscle deterioration [2]. Despite extensive research on therapeutic interventions for muscular dystrophy, no pharmacological treatment has proven consistently effective, making exercise therapy the primary intervention available [3]. Consequently, the development of novel therapeutic agents with minimal side effects and potential to prevent muscle wasting remains a critical area of research.

Although multiple biological alterations contribute to its progression, the precise mechanisms underlying skeletal muscle atrophy remain unclear. Disruptions in protein synthesis [4] and degradation [5], mitochondrial impairment [6], satellite cell dysfunction [7], elevated levels of reactive oxygen species (ROS) [8], and inflammatory processes [9] are all linked to this condition. The primary pathways governing protein synthesis in skeletal muscle involve phosphatidyl-inositol-3-kinase (PI3K) and Akt/mammalian target of rapamycin complex 1 (mTORC1) signaling, along with the inhibition of forkhead box protein O (FoxO) [10,11]. The activation of mTORC1 leads to the phosphorylation of p70S6 kinase 1 (p70S6K1) and eukaryotic translation initiation factor 4E-binding protein (4E-BP1), two key regulators of protein synthesis [10,11]. On the mitochondrial front, reduced biogenesis, increased ROS, and poor oxidative metabolism are notable in atrophic muscle [12,13]. AMP-activated protein kinase (AMPK) and sirtuin 1 (SIRT1) are known regulators of mitochondrial function, and peroxisome proliferator-activated receptor-gamma coactivator-1 α (PGC-1α) plays a key coordinating role in enhancing mitochondrial biogenesis in response to energy metabolism signals [14]. Therefore, targeting the AMPK/SIRT1/PGC-1α axis represents a potential strategy for mitigating muscle atrophy [15].

*Pueraria montana* var. *lobata* (PML), a member of the Fabaceae family, is a perennial vine with a long history of medicinal use in Northeast Asian countries including Japan, China, and Korea [16]. Known as ‘kudzu’ in Japan and ‘Gegen’ (*Radix Pueraria montana* var. *lobata*) in the Chinese pharmacopeia, PML has traditionally been used in the treatment of various ailments, such as fever, dysentery, hypertension, myocardial infarction, and arrhythmia [16,17]. Its cardiovascular benefits have led to its frequent incorporation in traditional medicine formulations [18]. Geographically, PML is native to East Asia and has also been introduced and naturalized in various other regions, including the southeastern United States, due to its rapid growth and environmental adaptability [19]. Furthermore, studies suggest that PML extract (PMLE) exhibits a range of pharmacological properties that may aid in managing liver disease, diabetes, osteoporosis, and neurological disorders [18,20,21,22]. Despite these therapeutic potentials, the impact of PMLE on muscle atrophy is yet to be thoroughly examined.

In this study, we evaluated the therapeutic efficacy of PMLE in both cellular and animal models of dexamethasone (DEX)-induced muscle atrophy, focusing specifically on its roles in stimulating protein synthesis and enhancing mitochondrial biogenesis through key signaling pathways.

## 2. Materials and Methods

### 2.1. Reagents and Antibodies

PMLE was obtained from the Korea Plant Extract Bank at the Korea Research Institute of Bioscience and Biotechnology (KRIBB, Cheongju, Korea). The extract was prepared using the leaf and stem parts of *Pueraria montana* var. *lobata*, which were harvested on 9 August 2007, during the mature vegetative growth stage. The botanical identity of the plant material was confirmed by expert taxonomists at KRIBB, and a voucher specimen was registered under the accession number KPM030-098. A 10 mg/mL stock was made in dimethyl sulfoxide (DMSO) and preserved at −20 °C. Essential reagents including TRIzol reagent (Cat. No. 15596026), Dulbecco’s modified Eagle’s medium (DMEM), fetal bovine serum (FBS), horse serum (HS), and antibiotics were sourced from Life Technologies (Carlsbad, CA, USA) and Gibco (Grand Island, NY, USA). Antibodies specific to myosin heavy chain (MyHC), myogenic differentiation 1 (MyoD), myogenin, PGC-1α, and SIRT1 were procured from Santa Cruz Biotechnology (Santa Cruz, CA, USA), while phosphorylation-specific antibodies for AMPK, AKT, mTOR, p70S6K1, and 4E-BP1, along with their total protein counterparts and glyceraldehyde 3-phosphate dehydrogenase (GAPDH), were obtained from Cell Signaling Technology (Danvers, MA, USA). Secondary antibodies, including horseradish peroxidase-conjugated anti-mouse and anti-rabbit IgG, were obtained from Enzo Life Sciences (Farmingdale, NY, USA).

### 2.2. Cell Culture and Differentiation Protocol

Mouse-derived C2C12 myoblasts (American Type Culture Collection; Manassas, VA, USA; ATCC-CRL 1772) were cultured in a growth medium (GM; DMEM containing 10% heat-inactivated FBS and antibiotics) at 37 °C under 5% CO_2_. For differentiation, cells were seeded in 6-well plates at a density of 5 × 10^4^ cells/well. Once 80–90% confluent, cells were transitioned from a growth medium to a differentiation medium (DM; DMEM with 2% HS) and incubated for 5–7 days (Control, CTL). PMLE was supplemented in the medium throughout the differentiation period with media refreshed every 2 days. Morphological assessment of myotubes was conducted via a phase-contrast microscope (Nikon TS2; Nikon, Tokyo, Japan). Photographs were captured at ×50.

### 2.3. Cell Viability Assessment

Cell viability was determined using the XTT (sodium 30-[1-(phenyl-aminocarbonyl)-3,4-tetrazolum]-bis(4-methoxy-6-nitro) benzenesulfonic acid hydrate and N-methyl dibenzopyrazine methyl sulfate) assay. C2C12 cells were plated in 96-well plates at a density of 1 × 10^3^ cells/well and treated with various concentrations of PMLE (0, 10, 50, or 100 ng/mL) during the differentiation period. At the endpoint, 50 µL of XTT reagent was added per well and incubated for 4 h. Absorbance was measured at 450 nm.

### 2.4. MyHC Immunofluorescence

Differentiated myotubes were fixed, permeabilized, and blocked using 3.7% paraformaldehyde, 0.1% Triton X-100, and 5% bovine serum albumin, respectively. The cells were incubated with a 1:200 dilution of anti-MyHC antibody at 4 °C for 24 h and subsequently with Alexa Fluor 488-conjugated secondary antibody (Life Technologies, Carlsbad, CA, USA). DAPI (4′,6-diamidino-2-phenylindole) was used for nuclear staining. Images were acquired using a fluorescence microscope (Nikon Eclipse Ts2-FL microscope; Nikon) and analyzed using ImageJ software version 1.54t (NIH, Bethesda, MD, USA).

### 2.5. Gene Expression Analysis via Quantitative Reverse Transcription Polymerase Chain Reaction (qRT-PCR)

Total RNA was isolated using TRIzol reagent (Thermo Fisher Scientific, MA, USA) and 1 μg was reverse transcribed into cDNA (RevertAid First Strand cDNA Synthesis Kit; Thermo Fisher Scientific). qRT-PCR was conducted using SYBR Green chemistry (SYBR Green Master Mix; Bioneer Co., Daejeon, Korea), and gene expression was normalized to GAPDH using the 2^−ΔΔCt^ method. Mouse primer sequences used were as follows: GAPDH: forward, 5′-TCAAGAAGGTGGTGAAGCAG-3′ and reverse, 5′-AGTGGGAGTTGCTGTTGAAGT-3′; MyHC: forward, 5′-GCCCAGTGGAGGACAAAATA-3′ and reverse, 5′-TCTACGTGCTCCTCAGCAT-3′; MyoD: forward, 5′-CGCTCCAACTGCTCTGATG-3′ and reverse, 5′-TAGTAGGCGGTGTCGTAGCC-3′; Myogenin: forward, 5′-CTACAGCTCCTTGCTCAGCTC-3′ and reverse, 5′-AGATTGTGGGCGTCTGTAGG-3′; PGC-1α: forward, 5′-CACCAAACCCACAGAAAACAG-3′ and reverse, 5′-GGGTCAGAGGAAGAGATAAAGTTG-3′; and SIRT-1: forward, 5′-GATCCTTCAGTGTCATGGTT-3′ and reverse, 5′-GAAGACAATCTCTGGCTTCA-3′. The amplification parameters were as follows: initial denaturation at 95 °C for 5 min followed by 40 cycles of 3-step PCR (denaturation at 95 °C for 1 min, annealing at 60 °C for 30 s, and a final extension at 72 °C for 1 min).

### 2.6. Protein Expression by Western Blotting

Following treatment, cells were rinsed with cold phosphate-buffered saline and lysed using RIPA buffer enhanced with protease and phosphatase inhibitors (Sigma-Aldrich; Saint Louis, MO, USA). Protein quantification was performed with the Bio-Rad DC Protein Assay Kit (Bio-Rad Laboratories, Hercules, CA, USA). Equal amounts (20–30 µg) of protein were electrophoresed on SDS-PAGE gels and transferred onto PVDF membranes (Millipore, Burlington, MA, USA). After blocking with 5% skim milk, membranes were probed with specific primary antibodies overnight at 4 °C. HRP-conjugated secondary antibodies were applied, and protein bands were visualized using a chemiluminescence detection system (Millipore). Densitometry was conducted using ImageJ software version 1.54t, with the results normalized to GAPDH levels.

### 2.7. Animal Model and Ethical Compliance

ICR male mice (8 weeks old) were obtained from Samtako Co., Ltd. (Osan, Republic of Korea) and acclimatized for one week. The animals were maintained under standard conditions (22–24 °C and 55–60% humidity in a room maintained on a 12/12 h light/dark cycle) with free access to water and a regular chow diet. All experiments were approved by the Institutional Animal Care and Use Committee (IACUC) of Wonkwang University (approval No. WKU21-68). Potential confounders were not specifically controlled in all animal studies. Mice were closely monitored throughout the experiment to assess their health status. During the experiment, all animals, experimental units, or data points were not excluded because the mice’s body weight, postoperative inflammatory response, and behavioral changes were not particularly evident.

### 2.8. Induction of Muscle Atrophy and Tissue Collection

Mice (n = 20, 33–36 g) were randomly allocated into four groups: CTL, DEX, DEX + PMLE, and PMLE (n = 6 each). DEX (25 mg/kg) was intraperitoneally administered for 8 consecutive days. PMLE (100 mg/kg) was orally administered during the same period. Control groups received vehicle solution (1% DMSO). Daily body weight was recorded. At study termination, mice were euthanized using ether anesthesia, and muscles [gastrocnemius (GAS), soleus (SOL), tibialis anterior (TA), extensor digitorum longus (EDL)] were collected, weighed, and either frozen for molecular assays or fixed for histological analysis.

### 2.9. Muscle Imaging via Micro-Computed Tomography (Micro-CT)

Muscle volume and density were assessed using a SkyScan1173 micro-CT scanner (SkyScn1173; Bruker CT, Kartuizersweg 3 B 2550; Kontich, Belgium). Samples were stabilized using parafilm and scanned at 130 kV and 66 µA. Cross-sectional reconstructions were performed using NRecon, and muscle tissue boundaries were defined at 270 ± 100 Hounsfield Units. Images were aligned and analyzed using DataViewer and CtAn software (version 1.5.2.4 64 bit; Bruker).

### 2.10. Histological Evaluation

GAS muscles fixed in formalin were embedded in paraffin and sectioned at 4 µm thickness. Sections were stained with hematoxylin and eosin to examine tissue morphology under light microscopy (Nikon TS2; Nikon).

### 2.11. Statistical Analysis

Sample sizes were determined a priori using G*Power software (version 3.1.9.7; Heinrich Heine University Düsseldorf, Düsseldorf, Germany). All experiments were replicated at least three times, and the data were reported as the mean ± standard deviation (SD) and analyzed using GraphPad Prism software (version 8.0, GraphPad Software, Inc., La Jolla, CA, USA). Statistical significance was determined using one-way analysis of variance (ANOVA) followed by Tukey’s multiple comparison test and Student’s *t*-test. Differences were considered statistically significant at *p* < 0.05. Prior to hypothesis testing, the assumptions of normality and homogeneity of variance were evaluated. Normality was assessed using the Shapiro–Wilk test, and homogeneity of variance was examined using Levene’s test. Residual diagnostics were also conducted to confirm linearity and overall model adequacy.

## 3. Results

### 3.1. PMLE Enhances Myogenic Differentiation

**Figure 1:**
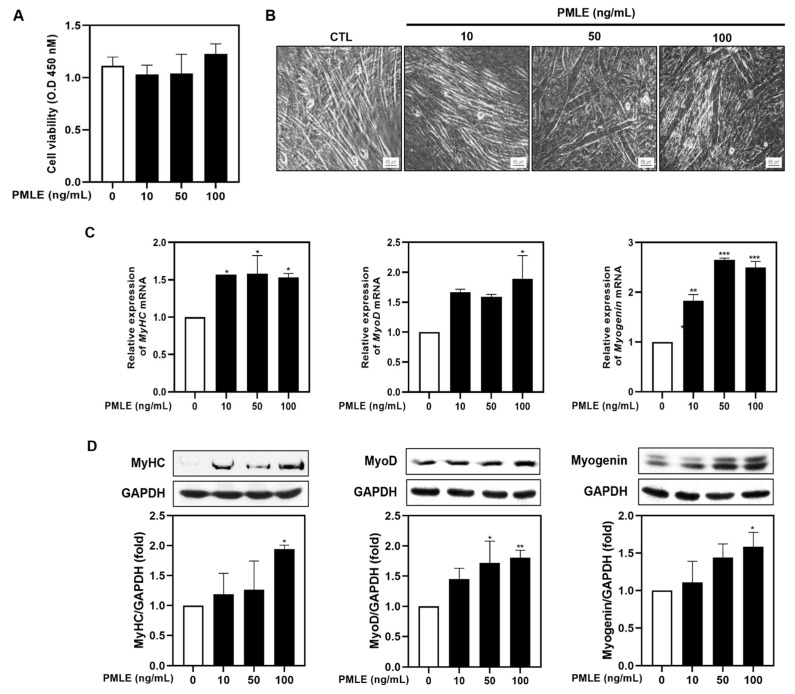
Effects of *Pueraria montana* var. *lobata* extract (PMLE) on myogenic differentiation in C2C12 myoblasts. (**A**) XTT assay showing no significant effect of PMLE (10, 50, 100 ng/mL) on C2C12 cell viability. Data are mean ± SD. (**B**) Phase-contrast images of myotube formation after PMLE treatment. Scale bar = 50 µm. (**C**) qRT-PCR analysis of *MyHC*, *MyoD*, and *myogenin* mRNA levels after PMLE treatment. Data are normalized to *GAPDH* and shown as fold change vs. control. (**D**) Western blot analysis of MyHC, MyoD, and myogenin protein levels. GAPDH was used as a loading control. Band intensities are shown as fold change vs. control. Statistical significance: * *p* < 0.05, ** *p* < 0.01, *** *p* < 0.001 vs. control.

To explore the impact of PMLE on muscle regeneration, we evaluated its influence on myogenic differentiation in C2C12 myoblasts. Cell viability was assessed via an XTT assay under differentiation conditions, and no cytotoxicity was observed across 10–100 ng/mL PMLE concentrations over 5 days (Figure 1A). Subsequent morphological evaluation revealed the formation of thick, spindle-shaped myotubes with multiple nuclear syncytia following PMLE treatment, particularly at 50 and 100 ng/mL (Figure 1B). These structures were more prominent than those in the untreated control group, suggesting an enhancement of myotube formation (Figure 1B). Quantitative RT-PCR (Figure 1D) and Western blot analyses (Figure 1D) demonstrated that PMLE significantly upregulated the expression of myogenic differentiation markers MyHC, MyoD, and myogenin in a dose–responsive manner, indicating a stimulatory effect on the differentiation process.

Overall, these results suggested that PMLE promoted myogenic differentiation by upregulating the expression of MyHC, MyoD, and myogenin.

### 3.2. PMLE Activates the Mitochondrial Biogenesis and Protein Synthesis Pathway

**Figure 2:**
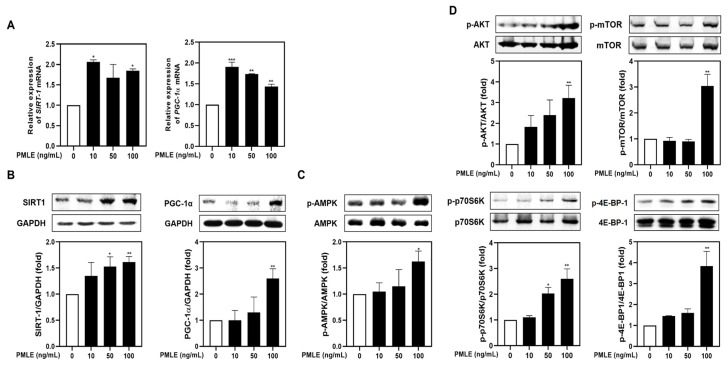
PMLE enhances mitochondrial biogenesis and protein synthesis signaling pathways in C2C12 myoblasts. (**A**) qRT-PCR analysis of SIRT1 and PGC-1α mRNA levels after PMLE (10, 50, 100 ng/mL) treatment. Data are normalized to GAPDH and shown as fold change vs. control. (**B**) Western blot analysis of SIRT1 and PGC-1α protein levels. GAPDH was used as a loading control. (**C**) Western blot of phosphorylated AMPK (p-AMPK); total AMPK was used as a control. (**D**) Western blot of p-AKT, p-mTOR, p-p70S6K, and p-4E-BP1; corresponding total proteins were used as loading controls. Band intensities are shown as fold change vs. control. Statistical significance: * *p* < 0.05, ** *p* < 0.01, *** *p* < 0.001 vs. control. PMLE, *Pueraria montana* var. *lobata* extract.

To determine whether PMLE modulates mitochondrial function during myogenic differentiation, we analyzed the mRNA and protein levels of PGC-1α and SIRT1. PMLE treatment resulted in a marked elevation of both markers, confirming the activation of mitochondrial biogenesis pathways (Figure 2A,B). In addition, PMLE enhanced AMPK phosphorylation, a key event in mitochondrial signaling (Figure 2C). Simultaneously, phosphorylation of proteins in the Akt/mTOR cascade—Akt, mTOR, p70S6K, and 4E-BP1—increased in a concentration-dependent manner (Figure 2D), suggesting improved protein synthesis capability. These data collectively support that PMLE facilitates muscle cell differentiation by concurrently activating mitochondrial biogenesis and protein synthesis pathways.

### 3.3. PMLE Reverses DEX-Induced Myotube Atrophy

**Figure 3:**
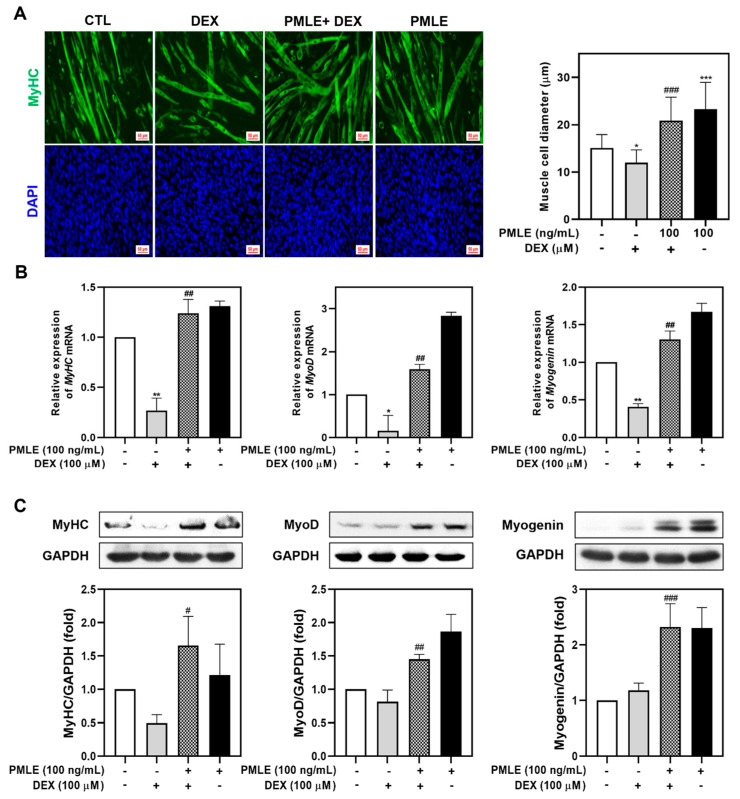
PMLE alleviates dexamethasone (DEX)-induced muscle atrophy and promotes myogenic differentiation in C2C12 myotubes. (**A**) Immunofluorescence staining of MyHC (green) and nuclei (DAPI, blue) in C2C12 myotubes treated with DEX (100 μM) and/or PMLE (100 ng/mL). The right panel shows the quantification of myotube diameter. (**B**) qRT-PCR analysis of *MyHC*, *MyoD*, and *myogenin* mRNA levels. Data are normalized to *GAPDH* and shown as fold change vs. control. (**C**) Western blot analysis of MyHC, MyoD, and myogenin protein levels. GAPDH was used as a loading control. Band intensities are shown as fold change vs. control. Statistical significance: * *p* < 0.05, ** *p* < 0.01, *** *p* < 0.001 vs. control group; ^#^ *p* < 0.05, ^##^ *p* < 0.01, ^###^ *p* < 0.001 vs. DEX-treated group. PMLE, *Pueraria montana* var. *lobata* extract.

We assessed PMLE’s ability to counteract DEX-induced muscle wasting in differentiated C2C12 myotubes. Treatment with 100 μM DEX significantly reduced myotube diameter, whereas PMLE co-treatment restored myotube size in a dose-dependent manner. (Figure 3A). The detrimental effects of DEX on myogenic marker expression were evident, with mRNA and protein levels of MyHC, MyoD, and myogenin declining. The co-administration of PMLE reversed these effects, significantly restoring both transcript and protein levels of these markers (Figure 3B,C). These findings indicated that PMLE effectively protected against DEX-induced muscle atrophy in C2C12 myotubes.

### 3.4. PMLE Restores the Mitochondrial Biogenesis and Protein Synthesis Pathway in DEX-Treated Myotubes

**Figure 4:**
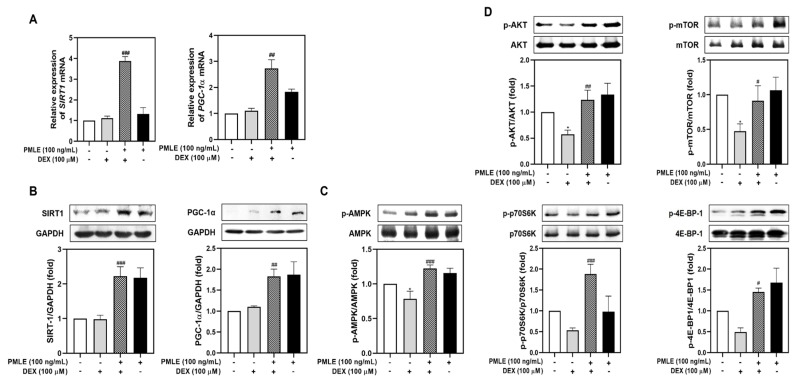
PMLE restores mitochondrial biogenesis and protein synthesis signaling pathways in DEX-induced muscle atrophy. (**A**) qRT-PCR analysis of *SIRT1* and *PGC-1α* mRNA levels in C2C12 myotubes treated with DEX (100 μM) and/or PMLE (100 ng/mL). Data are normalized to *GAPDH* and shown as fold change vs. control. (**B**) Western blot of SIRT1 and PGC-1α protein levels. GAPDH was used as a loading control. (**C**) Western blot of phosphorylated AMPK (p-AMPK); total AMPK was used as a control. (**D**) Western blot of p-AKT, p-mTOR, p-p70S6K, and p-4E-BP1; total proteins were used as loading controls. Band intensities are presented as fold change vs. control. Statistical significance: * *p* < 0.05 vs. control group; ^#^ *p* < 0.05, ^##^ *p* < 0.01, ^###^ *p* < 0.001 vs. DEX-treated group. PMLE, *Pueraria montana* var. *lobata* extract.

To explore the molecular mechanisms behind PMLE’s protective effects, we evaluated mitochondrial biogenesis and protein synthesis markers. Although DEX had a minimal effect on SIRT1 and PGC-1α, PMLE co-treatment led to significant upregulation of both genes and their protein products (Figure 4A,B). Next, we assessed the effects of PMLE on the AMPK signaling pathway, which is critical for mitigating mitochondrial oxidative damage in myotubes. As shown in Figure 4C, AMPK phosphorylation, slightly suppressed by DEX, was markedly elevated by PMLE. These results suggest that PMLE enhances mitochondrial biogenesis by promoting SIRT1 and PGC-1α expression through AMPK activation. Likewise, PMLE reactivated the Akt/mTOR axis, reversing the DEX-induced suppression of phosphorylated Akt, mTOR, p70S6K, and 4E-BP1 levels (Figure 4D). These findings indicate that PMLE enhances protein synthesis by activating the Akt/mTOR pathway.

### 3.5. PMLE Prevents Muscle Mass Loss in DEX-Treated Mice

**Figure 5:**
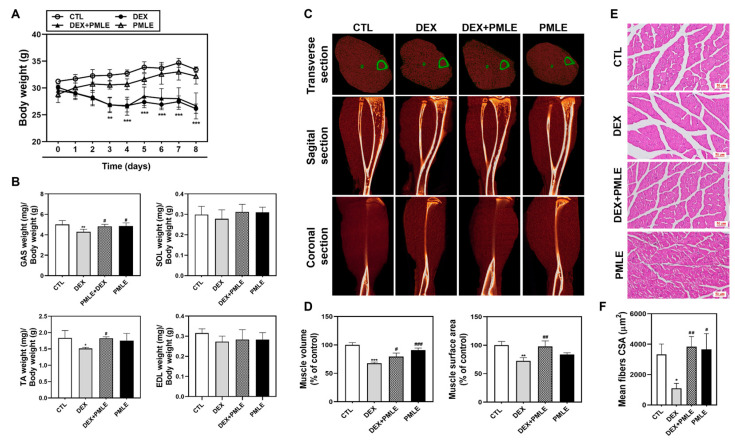
PMLE prevents DEX-induced muscle atrophy in vivo. (**A**) Body weight changes in mice treated with DEX (20 mg/kg) and/or PMLE (50 mg/kg) for 8 days. (**B**) Relative weights of GAS, SOL, TA, and EDL muscles normalized to body weight. (**C**) Micro-CT images (transverse, sagittal, coronal) of lower limb muscles. Green outlines indicate muscle boundaries. (**D**) Quantification of muscle volume and surface area (% of control). (**E**) H&E staining of GAS muscle cross-sections. Scale bar = 50 µm. (**F**) Quantitative analysis of myofiber CSA from histological images showing PMLE-mediated recovery of fiber size reduced by DEX treatment. Statistical significance: * *p* < 0.05, ** *p* < 0.01, *** *p* < 0.001 vs. control group; ^#^ *p* < 0.05, ^##^ *p* < 0.01, ^###^ *p* < 0.001 vs. DEX-treated group. PMLE, *Pueraria montana* var. *lobata* extract; CSA, cross-sectional area.

During the 8-day experiment, DEX-treated mice exhibited weight loss, while PMLE did not exacerbate this effect (Figure 5A). Muscle mass measurements showed that DEX notably reduced GAS and TA weights, whereas PMLE supplementation preserved muscle mass in these tissues (Figure 5B). Micro-CT imaging revealed that PMLE improved muscle volume and surface area, which were diminished by DEX (Figure 5C). Muscle characteristics were further quantified by analyzing the muscle distribution in the CT images. In the DEX group, calf muscle volume decreased by 32.2% (d = 2.23); however, PMLE treatment significantly increased calf muscle volume compared to that in the DEX group (Figure 5D). Similarly, calf muscle surface area, which was reduced by 27.6% (d = 0.95) by DEX, was improved by 25.2% (d = 4.20) in the DEX + PMLE 100 mg/kg group (Figure 5D). Histological analyses confirmed that PMLE alleviated structural damage in the muscle fibers, restoring compact, organized architecture (Figure 5E). Moreover, quantitative analysis of the muscle fiber cross-sectional area (CSA) revealed that DEX significantly reduced myofiber size, whereas PMLE treatment restored fiber diameter, indicating protective effects against muscle atrophy at the histological level (Figure 5F).

### 3.6. PMLE Enhances Myogenic Marker Expression In Vivo

**Figure 6:**
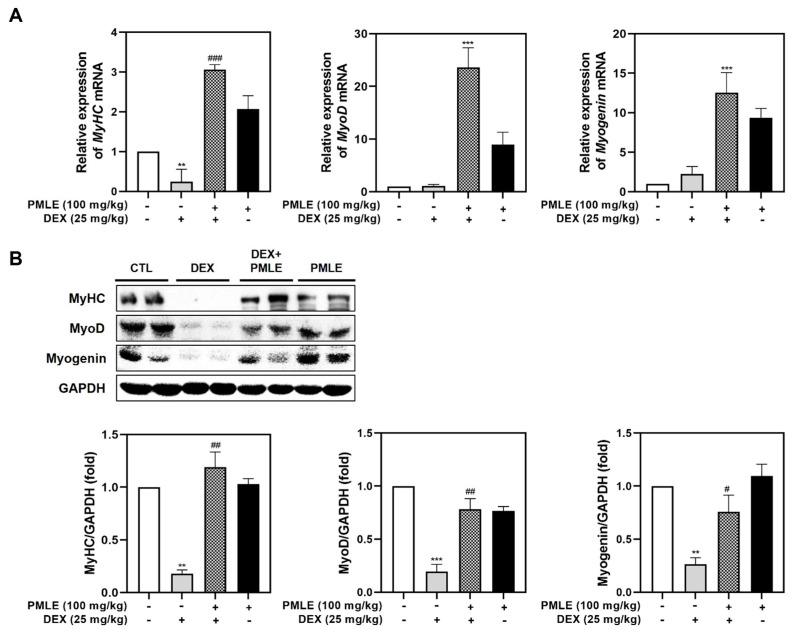
PMLE restores myogenic marker expression in DEX-induced muscle atrophy in vivo. (**A**) qRT-PCR analysis of *MyHC*, *MyoD*, and *myogenin* mRNA levels in gastrocnemius muscle from mice treated with DEX (25 mg/kg) and/or PMLE (100 mg/kg). Data are normalized to *GAPDH* and shown as fold change vs. control. (**B**) Western blot analysis of MyHC, MyoD, and myogenin protein levels in gastrocnemius muscle (CTL, DEX, DEX + PMLE, PMLE). GAPDH was used as a loading control. Band intensities are shown as fold change vs. control. Statistical significance: ** *p* < 0.01, *** *p* < 0.001 vs. control group; ^#^ *p* < 0.05, ^##^ *p* < 0.01, ^###^ *p* < 0.001 vs. DEX-treated group. PMLE, *Pueraria montana* var. *lobata* extract; DEX, dexamethasone; CTL, control.

In DEX-treated mice, PMLE significantly restored *MyHC* mRNA expression and also elevated *MyoD* and *myogenin* levels (Figure 6A). Western blot data mirrored these findings, with PMLE rescuing the protein expression of these markers suppressed by DEX (Figure 6B). These findings suggested that PMLE effectively counteracted DEX-induced muscle atrophy in mice by upregulating the expression of myogenesis-associated markers.

### 3.7. PMLE Actives Mitochondrial Biosynthesis Signaling in Atrophic Muscle

**Figure 7:**
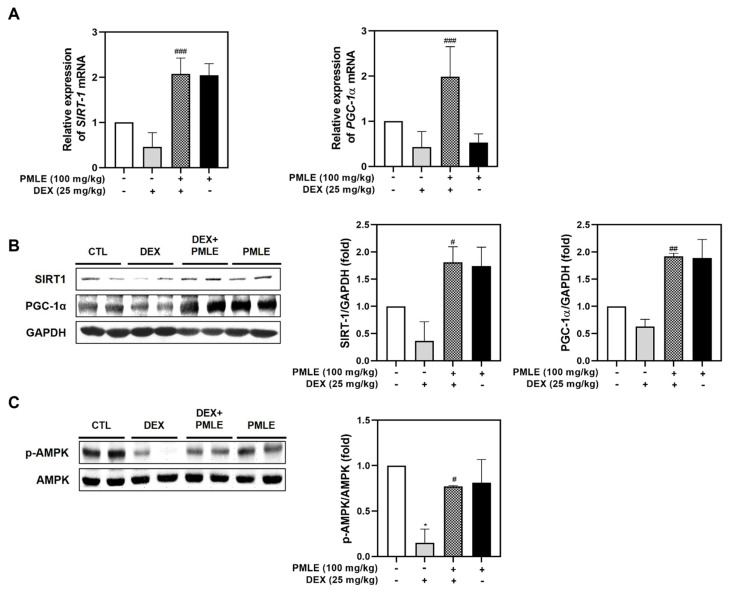
PMLE restores mitochondrial biogenesis and energy metabolism-related signaling pathways in DEX-induced muscle atrophy in vivo. (**A**) qRT-PCR analysis of *SIRT1* and *PGC-1α* mRNA levels in gastrocnemius muscle from mice treated with DEX (25 mg/kg) and/or PMLE (100 mg/kg). Data are normalized to *GAPDH* and shown as fold change vs. control. (**B**) Western blot analysis of SIRT1 and PGC-1α protein levels in each treatment group (CTL, DEX, DEX + PMLE, PMLE). GAPDH was used as a loading control. (**C**) Western blot of phosphorylated AMPK (p-AMPK); total AMPK used as a loading control. Band intensities are shown as fold change vs. control. Statistical significance: * *p* < 0.05 vs. control group; ^#^ *p* < 0.05, ^##^ *p* < 0.01, ^###^ *p* < 0.001 vs. DEX-treated group. PMLE, *Pueraria montana* var. *lobata* extract; DEX, dexamethasone; CTL, control.

In vivo analyses confirmed that PMLE enhanced the expression of mitochondrial biogenesis regulators. Both mRNA and protein levels of SIRT1 and PGC-1α were upregulated following PMLE treatment (Figure 7A,B). PMLE also significantly increased AMPK phosphorylation in skeletal muscle, corroborating its mitochondrial regulatory role observed in vitro (Figure 7C). These findings, consistent with the in vitro results, suggest that PMLE promotes mitochondrial biogenesis by upregulating SIRT-1 and PGC-1α expression through AMPK activation.

### 3.8. PMLE Stimulates Protein Synthesis via the Akt/mTOR Pathway In Vivo

**Figure 8:**
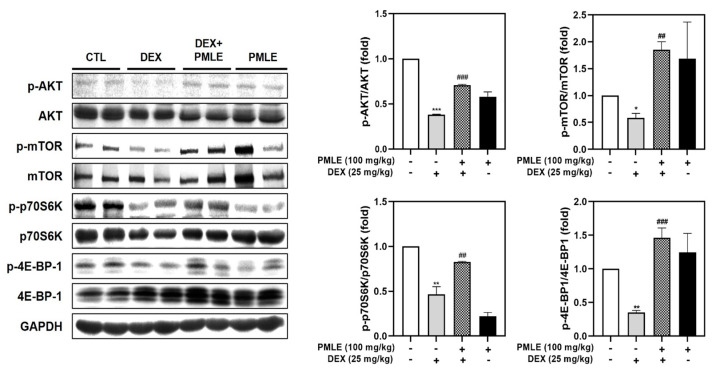
PMLE restores protein synthesis signaling pathways in DEX-induced muscle atrophy in vivo. Western blot analysis of phosphorylated and total AKT, mTOR, p70S6K, and 4E-BP1 in gastrocnemius muscle from mice treated with DEX (25 mg/kg) and/or PMLE (100 mg/kg). GAPDH was used as a loading control. Bar graphs (*right*) show the quantification of phosphorylated protein levels normalized to total protein levels. Statistical significance: * *p* < 0.05, ** *p* < 0.01, *** *p* < 0.001 vs. control group; ^##^ *p* < 0.01, ^###^ *p* < 0.001 vs. DEX-treated group. PMLE, *Pueraria montana* var. *lobata* extract; DEX, dexamethasone.

DEX treatment suppressed phosphorylation of critical protein synthesis mediators—Akt, mTOR, p70S6K, and 4E-BP1—without affecting total protein levels. PMLE effectively reversed these changes, restoring phosphorylation levels and suggesting a recovery of anabolic processes (Figure 8).

## 4. Discussion

This study demonstrates that PMLE promotes muscle cell differentiation and mitigates DEX-induced skeletal muscle atrophy through the activation of both anabolic and mitochondrial pathways. PMLE was non-toxic at concentrations below 100 ng/mL and significantly boosted the expression of key myogenic markers, such as MyHC, MyoD, and myogenin, while concurrently enhancing the Akt/mTOR and AMPK/SIRT1/PGC-1α signaling axes. The observed restoration of muscle mass and molecular signaling in vivo further corroborates the in vitro findings, highlighting PMLE’s dual action in both promoting differentiation and counteracting muscle degradation.

Excess glucocorticoids (GCs) are known to impair muscle protein synthesis by inducing atrophy-related factors, like myostatin [23,24]. These effects are frequently observed in chronic disease conditions and have been associated with oxidative damage and mitochondrial dysfunction [25,26]. DEX, a synthetic GC, has long been used in experimental models to simulate these muscle-wasting effects [27]. Consequently, DEX is widely used in both in vitro and in vivo models to study muscle atrophy, making the investigation of strategies to mitigate DEX-induced muscle loss critical for potential clinical applications. In our study, treatment with 100 μM DEX significantly reduced the width of myotubes formed by differentiated C2C12 cells, a result consistent with previous reports confirming the atrophy-inducing effects of DEX [28]. However, the co-administration of PMLE at a concentration of 100 ng/mL effectively restored myotube width (Figure 2A). These findings indicate that PMLE enhances myotube formation by facilitating myoblast fusion, which increases myotube diameter.

In this study, we demonstrated that PMLE significantly promotes myogenic differentiation by modulating key regulatory factors associated with muscle development. Specifically, PMLE enhanced the expression of MyHC1, MyoD, and myogenin, which play critical roles in muscle cell differentiation and maturation (Figure 1C,D). MyHC1, a major structural protein in mature muscle fibers, is directly associated with the contractile properties of myotubes. MyoD and myogenin, members of the myogenic regulatory factors family, orchestrate the transcriptional programs necessary for myoblast differentiation and fusion into multinucleated myotubes [29,30]. Numerous studies have established that MyoD acts as a master regulator of early myogenic commitment, promoting the expression of downstream differentiation markers including myogenin and MyHC isoforms [31]. Myogenin is essential for the late stages of myogenesis, driving the terminal differentiation process and ensuring proper muscle fiber formation [32]. PMLE treatment not only increases MyHC1 expression, thereby promoting the maturation of myotubes, but also enhances the expression of MyoD and myogenin, suggesting that PMLE comprehensively stimulates the entire muscle differentiation process. Furthermore, the ability of PMLE to upregulate these differentiation markers highlights its potential as a muscle regeneration promoter. By modulating MyHC1, MyoD, and myogenin expression, PMLE effectively supports both the early and late stages of myogenesis, suggesting that it may facilitate the repair and growth of muscle fibers under conditions of muscle damage or atrophy.

We also observed that PMLE promotes myogenic differentiation (Figure 2A–C) and inhibits DEX-induced muscle atrophy through the activation of the AMPK/SIRT1/PGC-1α signaling pathway (Figure 4A–C and Figure 7). This pathway is widely known as a key regulator of mitochondrial biogenesis and cellular energy homeostasis and is essential for muscle maintenance and regeneration. AMPK functions as an energy sensor activated under stress conditions, subsequently stimulating downstream targets, such as SIRT1 and PGC-1α [33]. SIRT1 is an NAD^+^-dependent deacetylase that plays a critical role in enhancing mitochondrial function and improving cellular resilience to stress [34]. Upon activation by AMPK, SIRT1 deacetylates and activates PGC-1α, a master regulator of mitochondrial biogenesis and oxidative metabolism [35]. PGC-1α, in turn, drives the transcription of genes involved in mitochondrial biogenesis, oxidative phosphorylation, and metabolic adaptation, thereby promoting muscle cell differentiation and improving muscle fiber quality [36]. In our study, PMLE treatment led to a significant increase in AMPK phosphorylation, which was accompanied by the upregulation of SIRT1 and PGC-1α expression (Figure 2A–C). These molecular changes were correlated with enhanced myotube formation and improved mitochondrial function, suggesting that the AMPK/SIRT1/PGC-1α axis is a primary mechanism by which PMLE exerts its beneficial effects. Furthermore, these findings are consistent with those of previous studies that have shown the involvement of this signaling pathway in protecting muscle cells from catabolic conditions and promoting an anabolic environment that supports muscle regeneration and growth.

The Akt/mTOR/p70S6K/4E-BP1 signaling pathway plays a central role in regulating protein synthesis and cell growth, both of which are critical for muscle maintenance and regeneration. Akt, a serine/threonine kinase, is a well-established upstream regulator that stimulates mTOR (mechanistic target of rapamycin) [37]. In turn, activated mTOR phosphorylates downstream effectors, such as p70S6K and 4E-BP1, leading to the increased translation of muscle-specific proteins and the promotion of myoblast differentiation into mature myotubes [37]. The ability of PMLE to enhance this signaling pathway was reflected in the observed increase in the phosphorylation of Akt, mTOR, p70S6K, and 4E-BP1 in treated myoblasts (Figure 2D). By stimulating these key nodes in the protein synthesis cascade, PMLE not only promotes myogenic differentiation but also supports the growth and maturation of myotubes. Furthermore, our results demonstrated that PMLE treatment effectively counteracted Akt/mTOR catabolic effects, restored signaling activity, and enhanced the differentiation process (Figure 4D and Figure 8). By reactivating the Akt/mTOR pathway and its downstream targets, PMLE contributes to the prevention of muscle degradation and the promotion of anabolic growth, highlighting its therapeutic potential in conditions characterized by muscle wasting.

In our in vivo experiments, DEX treatment significantly reduced body weight, which is consistent with the results of previous studies [28]. However, PMLE did not further reduce the body weight (Figure 5A), suggesting excellent biocompatibility at the tested concentrations. Fast-twitch muscles, such as the TA, are particularly vulnerable to glucocorticoid-induced atrophy because of their higher glucocorticoid receptor expression [38]. Accordingly, DEX reduced the weight of TA and GAS muscles, whereas PMLE effectively prevented this loss (Figure 5B). Micro-CT (Figure 5C,D) and histological analyses (Figure 5E) confirmed that PMLE alleviated the DEX-induced reduction in muscle cross-section area, consistent with improved muscle weights. At the molecular level, PMLE restored AMPK/SIRT1/PGC-1α and Akt/mTOR/p70S6K/4E-BP1 signaling pathways, which were suppressed by DEX. These results indicate that PMLE effectively mitigates DEX-induced muscle atrophy, both in terms of muscle mass preservation and the restoration of anabolic and mitochondrial biogenesis pathways. To better contextualize the therapeutic relevance of PMLE, it is worth noting that several natural compounds such as resveratrol, curcumin, and ginsenosides have also been reported to alleviate muscle atrophy through the modulation of similar pathways, including AMPK/SIRT1/PGC-1α and Akt/mTOR signaling axes [39,40,41]. Compared to these compounds, PMLE demonstrated comparable efficacy in restoring myogenic marker expression, promoting mitochondrial biogenesis, and enhancing protein synthesis, suggesting its potential as an alternative or complementary agent for muscle-wasting conditions. Also, while our findings support the therapeutic potential of PMLE in preventing muscle atrophy, it is important to consider potential safety concerns and limitations. Although PMLE has demonstrated cytocompatibility at concentrations used in this study and is derived from a traditionally consumed plant, comprehensive toxicity evaluations—particularly long-term and high-dose studies—are still limited. Previous research has reported that PMLE and its main isoflavones, such as puerarin, exhibit low toxicity in acute and subchronic models [42,43]. However, further preclinical studies focusing on chronic toxicity, pharmacokinetics, and possible off-target effects are necessary to ensure its safety for clinical applications. In addition, future studies should also assess sex-specific responses and potential interactions with existing medications.

## 5. Conclusions

This study demonstrated that PMLE effectively supports muscle health by promoting myogenic differentiation and counteracting DEX-induced muscle atrophy. By activating the AMPK/SIRT1/PGC-1α and Akt/mTOR pathways, PMLE enhances mitochondrial biogenesis, protein synthesis, and myotube formation, ultimately mitigating muscle degradation. These findings indicate that PMLE may serve as a promising therapeutic candidate for preventing and treating muscle-wasting disorders.

## Data Availability

The data used to support the findings of this study are available from the corresponding author upon request. The data are not publicly available due to institutional and ethical considerations related to the use of in vitro and in vivo experimental data. Although the dataset does not contain personally identifiable information, access is restricted to ensure compliance with ethical standards and institutional policies governing the use of biological research data.

## Abbreviations

The following abbreviations are used in this manuscript:

PMLE	*Pueraria montana* var. *lobata* extract
DEX	Dexamethasone
ROS	Reactive oxygen species
PI3K	Phosphatidyl-inositol-3-kinase
mTORC1	Akt/mammalian target of rapamycin complex 1
FoxO	Forkhead box protein O
p70S6K1	p70S6 kinase 1
4E-BP1	Factor 4E-binding protein
AMPK	AMP-activated protein kinase
SIRT1	Sirtuin 1
PGC-1α	Peroxisome proliferator-activated receptor-gamma coactivator-1 α
DMSO	Dimethyl sulfoxide
DMEM	Dulbecco’s modified Eagle’s medium
FBS	Fetal bovine serum
HS	Horse serum
MyHC	Myosin heavy chain
MyoD	Myogenic differentiation 1
GAPDH	Glyceraldehyde 3-phosphate dehydrogenase
GM	Growth medium
DM	Differentiation medium
DAPI	4′,6-diamidino-2-phenylindole
IACUC	Institutional Animal Care and Use Committee
GAS	Gastrocnemius
SOL	Soleus
TA	Tibialis anterior
EDL	Extensor digitorum longus
CT	Computed tomography
CTL	Control
XTT	Sodium 30-[1-(phenyl-aminocarbonyl)-3,4-tetrazolum]-bis(4-methoxy-6-nitro) benzenesulfonic acid hydrate and N-methyl dibenzopyrazine methyl sulfate
qRT-PCR	Quantitative reverse transcription polymerase chain reaction
GC	Glucocorticoid
CSA	Cross-sectional area
